# Associations of demographic, health, and risk-taking behaviors with tattooing in a population-based cross-sectional study of ~18,000 US adults

**DOI:** 10.21203/rs.3.rs-4838597/v1

**Published:** 2024-08-28

**Authors:** Rachel D McCarty, Britton Trabert, Morgan M Millar, David Kriebel, Laurie Grieshober, Mollie E Barnard, Lindsay J Collin, Katherine A Lawson-Michod, Brody Gibson, Jeffrey A Gilreath, Paul J Shami, Jennifer A Doherty

**Affiliations:** Huntsman Cancer Institute; Huntsman Cancer Institute; Huntsman Cancer Institute; University of Massachusetts Lowell; Huntsman Cancer Institute; Boston University; Huntsman Cancer Institute; Huntsman Cancer Institute; Huntsman Cancer Institute; Huntsman Cancer Institute; Huntsman Cancer Institute; Huntsman Cancer Institute

## Abstract

**Background:**

Little is known about current characteristics of individuals with tattoos. We quantified the prevalence of tattooing and associations of demographic, health, and risk-behavior factors with tattooing.

**Methods:**

We computed adjusted prevalence ratios (PR) of tattooing in a population-based analysis of > 18,000 Utah adults from the 2020–2021 Behavioral Risk Factor Surveillance System survey.

**Results:**

The prevalence of tattooing was 26% among women and 22% among men, with the highest prevalence among women ages 25–29 (45%). Tattoo prevalence was higher among younger individuals, individuals with a lower education level, and those without religious affiliation. Tattoo prevalence was higher among indviduals with current tobacco (women: PR = 2.89 [95% confidence interval (CI): 2.60, 3.20]; men: 3.39 [2.98, 3.86]), e-cigarette (women: 2.44 [2.21, 2.69]; men: 2.64 [2.37, 2.94]), and heavy alcohol use (women: 2.16 [1.93, 2.43]; men: 1.89 [1.63, 2.19]). Tattoo prevalence was lower among individuals receiving a flu (women: 0.84 [0.76, 0.92]; men: 0.75 [0.67, 0.84]) or COVID-19 vaccine (women: 0.65 [0.54, 0.79]; men: 0.75 [0.61, 0.92]).

**Conclusions:**

Several risk-taking behaviors were associated with tattooing. Tattoo studios/conventions may present opportunities for partnership with tobacco cessation, alcohol reduction, and vaccination public health initiatives.

## Background

Based on market and public opinion surveys, the prevalence of tattooing in the United States (US) has nearly doubled over the past 20 years with about 30% of adults estimated to have a tattoo.^1–4^ Tattooing holds cultural and personal significance, and motivations behind tattooing vary.^5^ Higher prevalence of tattooing has been observed among women,^1,2^ younger generations,^1,2,6^ individuals with no religious affiliation,^2,6^ and individuals with lower income or education.^2^

Prior studies among US adults have observed associations between risk-taking behaviors and tattooing.^6–8^ A 2004 study reported that tattooed individuals were more likely to have previously used alcohol or ever used recreational drugs.^6^ Recently, convenience-sampled surveys have described correlations between tattooing and tobacco use.^7,8^ Since tattoo prevalence has increased in recent years, the demographic, health, and lifestyle characteristics of the tattooed population may be changing. Obtaining up-to-date population-based estimates of tattooing prevalence and associations with demographic characteristics and health and risk behaviors is important for two reasons: first, because studies of associations between tattooing and health outcomes, such as cutaneous conditions^9^ or cancer,^10^ must carefully collect data on and account for potential confounders; and second, because partnering with tattoo studios and conventions to employ public health interventions might be an effective way to reach at-risk individuals.

We leveraged the 2020–2021 population-based Utah Behavioral Risk Factor Surveillance System (BRFSS) telephone survey of over 18,000 Utah adults^11,12^ to quantify the prevalence of tattooing by demographic factors, and to characterize associations of health and risk-taking behaviors with tattooing.

## Methods

The BRFSS survey of health-related behaviors is administered by every state in the US and uses a disproportionate stratified sampling design which stratifies by phone type (listed numbers, unlisted numbers, and cell phones) and region.^13^ A two-part weighting methodology consisting of design weights and iterative proportional fitting (i.e., raking), ensures the sample is reflective of the target population. The current cross-sectional study includes data from the 2020–2021 Utah BRFSS survey, with response proportions of 55% in 2020 and 47% in 2021 (n = 21,542).^11,12^

Individual states may add questions to the BRFSS. We added three tattoo questions to the Utah survey that we developed and piloted: 1) What is the total number of tattooing sessions you have had? 2) How many of your tattoos are bigger than your palm? and 3) How old were you when you got your first tattoo? Participants were asked to include every tattoo they had ever received using a tattoo machine, even if it was faded, covered up, or had been removed. Cosmetic tattoos were not included as they are typically applied with handheld tools that deposit pigments at a shallower depth and are semi-permanent.^14^ We excluded 2,855 individuals missing answers to all tattoo questions, for an analytic dataset of 18,687 individuals.

We defined “ever tattooed” as one or more tattoo sessions, and “never tattooed” as no tattoo sessions. We calculated tattoo prevalence by demographic characteristics (i.e., sex, race and ethnicity, age, marital status, religious affiliation, education, sexual orientation) by computing unweighted counts, and weighted proportions, accounting for the survey design. We also characterized the number of tattoo sessions, number of large tattoos, and age at first tattoo. We stratified by sex due to differences in tattoo prevalence and health and risk-behaviors. We also stratified by affiliation with the Church of Jesus Christ of Latter-day Saints (LDS), as it is the predominant religion in Utah^15^ and has historically discouraged tattoos, tobacco use, and alcohol use.^16^

Data on risk-taking behaviors was also obtained from the BRFSS. Variables of interest included: former and current tobacco smoking; current electronic cigarette (e-cigarette) use; binge drinking within the past 30 days (4 + drinks for women/5 + drinks for men); heavy drinking within the past 30 days (7 + drinks per week for women/14 + drinks per week for men); marijuana use within the past 30 days; reason for marijuana use. We also evaluated health-related access and behaviors queried on the BRFSS, including: current health insurance (yes/no); instances in the past 12 months when individuals were unable to see a doctor due to cost; mammograms within the past two years (among women ages 40 +^17^); pap testing within the past three years (among women ages 21–65 who had not had a hysterectomy^18^); ever had a human papillomavirus (HPV) test (among women ages 21–65^18^); ever had a prostate-specific antigen (PSA) test (among men ages 40+); ever had a human immunodeficiency virus (HIV) test; had a colonoscopy within the past 10 years (among ages 50–75^19^); had a flu vaccine in the past 12 months; up-to-date on vaccines (excluding flu and COVID-19); received at least one dose of COVID-19 vaccine or intend to.

We fit quasi-Poisson models, which account for overdispersion,^20^ to compute prevalence ratios (PRs) and 95% confidence intervals (CIs) for each demographic, risk-taking, and health-related access and behavior variable and prevalence of tattooing. Multivariable models adjusted for age, race and ethnicity, and education level, and were stratified by sex and LDS vs. non-LDS affiliation. All analyses were conducted using R Statistical Software (v4.3.1; R core team 2023; Vienna, Austria).

## Results

### Demographics

The prevalence of tattooing was 26% among women and 22% among men (Table 1). NH American Indian or Alaskan Native and NH multiracial women had over 30% higher tattoo prevalence compared with NH White women (PR=1.34 [95% CI: 1.01, 1.77] and 1.36 [1.01, 1.84] respectively) (Table 2). NH American Indian or Alaskan Native and NH multiracial men had roughly 60% higher prevalence than NH White men (1.64 [1.15, 2.36] and 1.57 [1.12, 2.20] respectively). NH Pacific Islander women and men had increased tattoo prevalence (women: 1.28 [0.83, 1.98]; men: 1.28 [0.78, 2.09])). Women ages 25–29 had a 45% prevalence of tattooing, over five times higher than those ages 60 and older (5.21 [4.39, 6.19]), while men 25–29 had over three times the prevalence of men ages 60 and older (3.42 [2.81, 4.17]). Being unmarried was associated with 60% higher prevalence of tattooing among women (1.62 [1.47, 1.78]) and 30% higher prevalence among men (1.34 [1.20, 1.49]) compared with married individuals. Women with less than a high school diploma/General Educational Diploma (GED) had 90% higher tattoo prevalence (1.90 [1.54, 2.35]), while men with less than a high school diploma/GED had three times higher prevalence (3.04 [2.47, 3.74]) than those with a four-year college degree. Individuals identifying as a sexual minority (gay, bisexual, or other) had a two-fold higher prevalence of tattooing among women and a 24% higher prevalence of tattooing among men than individuals identifying as straight (women: 2.05 [1.84, 2.28]; men: 1.24 [1.04, 1.49]).

The prevalence of tattooing differed dramatically by LDS status; the prevalence was 44% in non-LDS women and 35% in non-LDS men, versus 10% in LDS women and 9% in LDS men (Table 1). LDS women and men had roughly a 75% decreased prevalence of tattooing (women: 0.23 [0.20, 0.26]; men: 0.27 [0.24, 0.32]) compared with those without religious affiliation (Table 2). Associations were weaker for Protestant (0.76 [0.67, 0.86]) and Catholic (0.64 [0.53, 0.78]) affiliation among women; no associations with these affiliations were observed among men.

With respect to the more detailed tattooing exposures, 10% of women and 9% of men had 4 or more tattoo sessions (Supp Table 2). Among both women and men, 15% had at least one tattoo larger than their palm; and 12% of women and 11% of men received their first tattoo at age 19 or younger.

### Risk-taking behaviors

Compared with never use, both former and current tobacco smoking were associated with increased tattoo prevalence among women (former: 2.73 [2.50, 2.99]; current: 2.89 [2.60, 3.20]) and men (former: 2.80 [2.49, 3.14]; current: 3.39 [2.98, 3.86]). Associations were most pronounced among LDS women (former: 4.60 [3.59, 5.90]; current: 5.74 [4.35, 7.57]) and LDS men (former: 4.30 [3.19, 5.81]; current 6.47 [4.49, 9.33]) (Table 3).

Patterns were similar for e-cigarette use; current use vs no current use was associated with increased tattoo prevalence among both women (2.44 [2.21, 2.69]) and men (2.64 [2.37, 2.94]), particularly for LDS women (4.65 [3.35, 6.46]) and men (5.73 [4.04, 8.13]) (Table 3).

Binge drinking and heavy drinking within the past 30 days were associated with tattooing among women (binge: 2.19 [1.99, 2.40]; heavy: 2.16 [1.93, 2.43]) and men (binge: 2.15 [1.93, 2.38]; heavy: 1.89 [1.63, 2.19]) particularly among LDS women (binge: 4.14 [2.78, 6.16]; heavy: 5.51 [3.79, 8.01]) and LDS men (binge: 3.73 [2.69, 5.17]; heavy: 3.20 [2.04, 5.02]).

Marjiuana use within the past 30 days was associated with tattooing among women (2.10 [1.89, 2.34]) and men (2.12 [1.89, 2.37]). Again, associations were strongest among LDS women (3.82 [2.77, 5.27]) and LDS men (3.28 [2.17, 4.96]). Among non-LDS women, tattooing was most associated with both medical and non-medical use (1.61 [1.43, 1.80]). Among non-LDS men, tattooing was most associated with medical use only (1.55 [1.29, 1.86]) and medical and non-medical use (1.57 [1.36, 1.82]).

### Health-seeking behaviors

Having health insurance compared with no insurance was associated with decreased tattoo prevalence among LDS women (0.62 [0.44, 0.87]), while among non-LDS women, it was associated with increased prevalence (1.24 [1.05, 1.47]). Patterns were similar among men; LDS men with health insurance had decreased prevalence (0.60 [0.42, 0.87]) while non-LDS men had increased prevalence of tattooing (1.12 [0.95, 1.32]) (Table 4).

Inability to see a doctor at least once in the past 12 months due to cost was associated with tattooing among both women (1.32 [1.19, 1.48]) and men (1.21 [1.05, 1.39]). Associations were most pronounced among LDS women (1.83 [1.38, 2.44]), and men (1.29 [0.81, 2.06]), while no associations were observed among non-LDS women and men.

Having had a pap test within the past three years was associated with tattooing among women (1.38 [1.16, 1.64]), with similar results regardless of LDS affiliation. Ever vs never having had an HPV test was associated with tattooing among women overall (1.65 [1.41, 1.93]), with the most pronounced association among LDS women (1.92 [1.34, 2.76]). Ever having had an HIV test was associated with increased tattoo prevalence among both women (1.93 [1.76, 2.12]) and men (1.92 [1.73, 2.12]), with the most pronounced association among LDS women (2.59 [2.03, 3.30]). Associations of mammography within the past two years, ever having a PSA test, or having a colonoscopy within the past 10 years with tattooing were near-null.

Associations between vaccinations and tattooing varied. Receiving a flu vaccine in the past 12 months was associated with lower tattoo prevalence among women (0.84 [0.76, 0.92]) and men (0.75 [0.67, 0.84]). No associations were observed between being up-to-date on all vaccines and tattooing among women, however among men this was associated with decreased tattooing overall (0.81 [0.69, 0.94]), especially among LDS men (0.67 [0.45, 1.00]). Receiving at least one dose of a COVID-19 vaccine or intending to was assocated with lower tattoo prevalence among women (0.65 [0.54, 0.79]) and men (0.75 [0.61, 0.92]), with the most pronounced association among LDS women (0.37 [0.24, 0.58]), and no association among LDS men (1.11 [0.58, 2.13]).

## Discussion

To our knowledge, this is the largest US-based study to date to characterize relationships of demographic, health, and risk-taking factors with tattooing. While we observed that the overall prevalence of tattooing is lower in Utah than that reported in national market/public opinion surveys,^1–4^ the prevalence of tattooing among non-LDS individuals in Utah was higher than that reported in those surveys. Consistent with prior studies, we observed higher tattoo prevalence among women,^1,2^ younger individuals,^1,2,6^ individuals with less education,^2^ and individuals without religious affiliation.^2,6^ The high prevalence of tattooing in younger age groups and early age at tattooing observed in our and other studies as well as the increasing prevalence of tattooing^1,4^ highlights the need to characterize factors associated with tattooing.

We observed variations in tattoo prevalence by race and ethnicity, with higher prevalences among NH American Indian or Alaskan Native and NH Pacific Islander compared with NH White individuals, which have not been previously reported. We observed lower prevalences among NH Asian individuals compared with NH White individuals, which is similar to findings from the 2023 Pew Research Center survey.^2^ However, we observed lower prevalence of tattooing among NH Black compared with NH White individuals, while the Pew survey reported higher prevalence among Black individuals.^2^

Our study supports evidence that tobacco, heavy alcohol, and marijuana use are associated with tattooing. Associations of tobacco and heavy alcohol use with tattooing were previously reported in a study of military recruits interviewed in 1999^21^ and a 2016 survey via Amazon’s Mechanical Turk, a crowdsourced online platform (for smoking only; they did not examine alcohol use).^8^ In the only prior population-based study, which was conducted in 2004, past drinking and recreational drug use were more prevalent among tattooed individuals.^6^ This study is not directly comparable to ours as they did not examine heavy drinking or marijuana specifically. We also report the novel findings that e-cigarette use and lack of flu or COVID-19 vaccination were associated with tattooing.

In our study, we observed a lower prevalence of tattooing among individuals with certain religious affiliations, which is consistent with findings reported in the 2004 study.^6^ However, in our study, we were able to assess the associations by sex. The prevalence of tattooing was considerably lower among LDS women and men compared with those who were non-LDS, which we expected as the LDS church has historically discouraged members from getting tattoos. We also observed a lower prevalence of tattooing among women, but not men, who identified as Protestant or Catholic compared with those with no religion.

Associations between barriers to healthcare access (lack of health insurance and inability to see a doctor due to cost) and tattooing were observed only among LDS individuals, and associations between several risk-taking behaviors and tattooing were stronger for LDS individuals compared with non-LDS individuals. Reasons for this are likely multifactorial as the associations between risk-taking behaviors, mental health, and social determinants of health are complexly interconnected.

### Strengths and Limitations

A limitation of this study is the potential for recall bias as individuals may misremember the number of tattoo sessions or age at first tattoo. However, as tattooing is permanent and our main analyses focused on ever/never tattooed, recall bias was likely minimal and non-differential across different demographic, health, and risk-taking behaviors. Further, it is unclear the degree to which the associations observed in this study are generalizable to other US states, because of the high percentage of Utah residents who are members of the LDS church (~ 50% of study participants). Despite these limitations, this population-based study is the largest to date providing the most current comprehensive characterization of detailed demographic and health and risk behaviors among tattooed individuals.

## Conclusions

Tattooing, which holds importance both culturally and as an artistic medium for self-expression, is an exposure with particularly high prevalence among women, younger generations, individuals with less education, and individuals without a religious affiliation. Several risk-taking behaviors, including tobacco, e-cigarette, heavy alcohol, and marijuana use are associated with tattooing, as is decreased adherence to flu and COVID-19 vaccine recommendations. Public health entities may consider partnering with tattoo studios and tattoo conventions with tobacco cessation, alcohol reduction, and vaccine initiatives in order to reach individuals with greater need.

## Data Availability

The 2020 and 2021 BRFSS data used in this study are available from the Utah Department of Health and Human Services. Restrictions apply to the availability of these data.

## Abbreviations

BRFSS: Behavioral Risk Factor Surveillance System
CI: confidence interval
e-cigarette: electronic cigarette
GED: General Educational Diploma
HIV: human immunodeficiency virus
HPV: human papilomavirus
LDS: Church of Jesus Christ of Latter-day Saints
NH: non-Hispanic
PR: prevalence ratio
PSA: prostate-specific antigen
US: United States
